# Integrated analysis of genome-wide miRNAs and targeted gene expression in esophageal squamous cell carcinoma (ESCC) and relation to prognosis

**DOI:** 10.1186/s12885-020-06901-6

**Published:** 2020-05-06

**Authors:** Howard Yang, Hua Su, Nan Hu, Chaoyu Wang, Lemin Wang, Carol Giffen, Alisa M. Goldstein, Maxwell P. Lee, Philip R. Taylor

**Affiliations:** 1 Center for Cancer Research, NCI, Bethesda, MD 20892 USA; 2 Leidos Biomedical Research, Inc., Frederick, MD 21702-1201 USA; 3 Division of Cancer Epidemiology and Genetics, NCI, Bethesda, MD 20892 USA; 4 Information Management Services, Inc, Calverton, MD 20705 USA

**Keywords:** Esophageal squamous cell carcinoma, microRNAs, mRNAs, Prognosis

## Abstract

**Background:**

Esophageal squamous cell carcinoma (ESCC) is a leading cause of cancer death worldwide and in China. We know miRNAs influence gene expression in tumorigenesis, but it is unclear how miRNAs affect gene expression or influence survival at the genome-wide level in ESCC.

**Methods:**

We performed miRNA and mRNA expression arrays in 113 ESCC cases with tumor/normal matched tissues to identify dysregulated miRNAs, to correlate miRNA and mRNA expressions, and to relate miRNA and mRNA expression changes to survival and clinical characteristics.

**Results:**

Thirty-nine miRNAs were identified whose tumor/normal tissue expression ratios showed dysregulation (28 down- and 11 up-regulated by at least two-fold with *P* < 1.92E-04), including several not previously reported in ESCC (miR-885-5p, miR-140-3p, miR-708, miR-639, miR-596). Expressions of 16 miRNAs were highly correlated with expressions of 195 genes (*P* < 8.42E-09; absolute rho values 0.51–0.64). Increased expressions of miRNA in tumor tissue for both miR-30e* and miR-124 were associated with increased survival (*P* < 0.05). Similarly, nine probes in eight of 818 dysregulated genes had RNA expression levels that were nominally associated with survival, including *NF1, ASXL1, HSPA4, TGOLN2, BAIAP2, EZH2, CHAF1A, SUPT7L*.

**Conclusions:**

Our characterization and integrated analysis of genome-wide miRNA and gene expression in ESCC provides insights into the expression of miRNAs and their relation to regulation of RNA targets in ESCC tumorigenesis, and suggest opportunities for the future development of miRs and mRNAs as biomarkers for early detection, diagnosis, and prognosis in ESCC.

## Background

Esophageal carcinoma occurs worldwide as the sixth leading cause of cancer mortality [1] and is an aggressive tumor with a 5-year survival rate less than 20%, due largely to late diagnosis [2]. It is the fourth most common new cancer in China [3], and Shanxi Province in north central China has some of the highest esophageal cancer rates in the world [4, 5]. Improved understanding of the molecular mechanisms underlying esophageal carcinogenesis and its molecular pathology should help identify new biomarkers for early detection strategies that reduce esophageal squamous cell carcinoma (ESCC) mortality.

Gene expression profiling can improve our understanding of molecular alterations during carcinogenesis. Biomarkers of these molecular alterations, in turn, may be useful in diagnosing cancers, particularly early, curable cancers. They may also identify druggable targets for therapy or be useful in predicting prognosis. Regulatory mechanisms underlying gene expression are vital functions in biological processes. The discovery of microRNAs (miRNAs) has revealed a hidden layer of gene regulation that can tie multiple genes together into biological networks. More than 2500 mature human miRNAs have been identified thus far (miRBase assembly version GRCh38) [6] since they were first described in 1993 [7]. Studies have demonstrated that miRNAs modulate gene expression by binding to the 3′ untranslated region (UTR) of target mRNAs, causing either mRNA degradation or translational inhibition [8, 9]. It is also known that a single miRNA can regulate many mRNAs, and that one mRNA can be influenced by many miRNAs. While RT-PCR is typically used to study a few candidate target miRNAs, DNA microarrays and next-generation sequencing are techniques that enable studies at the genome-wide scale level. Using these techniques, miRNA and mRNA profiling has been reported for numerous cancers (e.g., lung, breast, stomach, prostate, colon, pancreas, hepatocellular carcinoma, ESCC) using a variety of biosample types (ie, frozen tissue, formal fixed paraffin embedded, whole blood, serum, plasma [10, 11]) with results relatable to several patient outcomes such as diagnosis, prognosis, and prediction.

Thus far there have been only a few reports of genome-wide analyses of both miRNA and mRNA expression in paired tumor/normal tissues from ESCC patients, but these studies have included only a small number of cases [12] or very limited numbers of patient-paired samples [13]. Several groups from Japan have performed miRNA expression profiles in serum samples to search for biomarkers useful in clinical diagnosis or prognosis [11, 14–17], while others have applied DNA microarray analysis to discrete numbers of paired ESCC tissue samples for miRNA profiling only [18–23]. Herein we report a genome-wide study of both miRNA and mRNA profiles performed in frozen, paired tumor/normal tissues from 113 ESCC cases to identify dysregulated miRNAs, correlate miRNA and gene expression, and relate miRNA and mRNA expression with clinical characteristics, including survival.

## Methods

### Study population

Patients enrolled in the project included consecutive cases of ESCC who presented to.

the Surgery Department of the Shanxi Cancer Hospital in Taiyuan, Shanxi Province, PR China, between 1998 and 2003, who had no prior therapy for their cancer, and who underwent surgical resection of their tumor at the time of their hospitalization. After obtaining written informed consent, patients were interviewed to obtain information on demographic and lifestyle cancer risk factors, and family history of cancers. Clinical data were collected at the time of hospitalization (between 1998 and 2003) and cases were followed after surgery for up to 69 months to ascertain vital status (median follow-up 23 months). In total, 113 ESCC cases were evaluated in the present study. All cases were histologically confirmed as ESCC by pathologists at both the Shanxi Cancer Hospital and the National Cancer Institute (NCI). This study was approved by the Institutional Review Boards of the Shanxi Cancer Hospital and the NCI.

### Tissue collection and total RNA preparation

Paired esophageal cancer and normal tissue distant to the tumor were collected during surgery. Tissues for RNA analyses were snap frozen in liquid nitrogen and stored at − 130 °C until used. Selection of patients for RNA studies was based solely on the availability of appropriate tissues for RNA testing (ie, consecutive testing of cases with available frozen tissue, tumor samples that were predominantly (> 50%) tumor, and tissue RNA quality/quantity adequate for testing). Total RNA was extracted by two methods: one was extracted by the Trizol method following the protocol of the manufacturer (http://tools.invitrogen.com/content/sfs/manuals/trizol_reagent.pdf). A second method of RNA extraction was by using Allprep RNA/DNA/Protein mini kit from Qiagen, following the manufacturer’s instructions (http://www.qiagen.com/literature/render.aspx?id=2067). For both extraction methods, the quality and quantity of total RNA were determined on the RNA 6000 Labchip/Agilent 2100 Bioanalyzer (Agilent Technology, Inc.).

### ABI miRNA expression array by RT-PCR

TaqMan® Low Density Arrays were used to measure MicroRNA expression. Analyses were performed using a 9700HT fast real-time PCR system from ABI. Comprehensive coverage of Sanger miRBase v14 was enabled across a two-card set of TaqMan® Array MicroRNA Cards (Cards A and B) for a total of 664 unique human miRNAs. In addition, each card contained one selected endogenous control assay (MammU6) printed four times, 5 endogenous gene probes (RNU 24, 43, 44, 48, 6B), and one negative control assay (ath-miR159a). Card A focused on more highly characterized miRNAs, while Card B contained many of the more recently discovered miRNAs along with the miR* sequences.

The protocol was according to the manufacturer’s manual at http://www3.appliedbiosystems.com/cms/groups/mcb_support/documents/generaldocuments/cms_042167.pdf. Briefly, three microliter (ul) of total RNA (350–1000 ng) was added to 4.5uL of RT reaction mix, which consisted of 10x Megaplex RT Primers, 100 mM dNTPs with dTTP, 50 U/uL MultiScribe Reverse Transcriptase, 10x RT buffer, 25 mM MgCl_2_, 20 U/uL RNase Inhibitor, and nuclease-free H_2_O. The samples were run on a thermal cycler using the following conditions: 40 cycles of 16 °C for 2 min, 42 °C for 1 min, and 50 °C for 1 s. All reactions were completed with a final incubation at 85 °C for 5 min. Six microliters of cDNA generated were mixed with 450uL of 2x TaqMan Universal PCR Master Mix with no AmpErase UNG, and 444uL of nuclease-free H_2_O. 100uL of the reaction mix was added to each of 8 fill ports on a TaqMan MicroRNA Array. The filled Array was centrifuged twice at 1200 rpm for 1 min, and then sealed with 8 fill ports film. Arrays were run on a 7900HT RT-PCR System with the SDS software and the comparative CT method was used to determine the expression levels of mature miRNAs.

### Probe preparation and hybridization for mRNA microarrays

Of the 113 paired ESCC samples, 34 pairs were run on Human U133A chips, 73 pairs on U133A_2 chips, and 6 pairs on U133Plus_2 chips from Affymetrix. Probes were prepared according to the protocol provided by the manufacturer (Affymetrix Genechip expression analysis technical manual), available from: http://www.affymetrix.com/support/index.affx).

Procedures included first strand synthesis, second strand synthesis, double-strand cDNA cleanup, in vitro transcription, cRNA purification, and fragmentation. Twenty micrograms of biotinylated cRNA were finally applied to the hybridization arrays of the Affymetrix GeneChip. After hybridization at 45 °C overnight, arrays were developed with phycoerythrin-conjugated streptavidin by using a fluidics station (Genechip Fluidics Station 450) and scanned (Genechip Scanner 3000) to obtain quantitative gene expression levels. Paired tumor and normal tissue specimens from each patient were processed simultaneously during the RNA extractions and hybridizations.

### ABI miRNA expression array data analysis

RQ Manager integrated software from the ABI was used to normalize the entire signal generated. Expression levels (as fold changes, or FC) were calculated when both tumor and normal sample gave signals in the assays using DataAssist software v2.0 (Life Technologies, http://www.lifetechnologies.com/about-life-technologies.html). The miRNAs that showed signals in tumor only or normal only were dropped from further analysis. In the present study, the data are presented as fold change calculated using the 2 ^-ΔΔCT^ method. Results of the real-time PCR data were represented as C_T_ values, where C_T_ was defined as the threshold cycle number of PCRs at which amplified product was first detected. The average C_T_ was calculated for both the target genes and MammU6, and the ΔC_T_ was determined as the mean of the C_T_ values for the target gene minus the mean of the quadruplicate C_T_ values for MammU6. The ΔΔC_T_ represented the difference between the paired tissue samples, as calculated by the formula ΔΔC_T_ **=** (ΔC_T_ of tumor - ΔC_T_ of normal). The N-fold differential expression in the target gene of a tumor sample compared to the normal sample counterpart was expressed as 2 ^-ΔΔCT^.

As our normalization procedure was based on MammU6, our endogenous control, we assessed the technical variation of our normalization procedure by determining the coefficient of variation (CV) of the quadruplicate C_T_ values for MammU6. CVs (standard deviation divided by mean) were calculated for each case separately for the 113 normal and 113 tumor tissue samples tested. Over all samples, CVs for MammU6 were determined to be very low – 1.3% for normal tissues and 0.7% for tumor tissues, indicating that technical variation was minimal; thus, reproducibility was excellent for use of MammU6 in our normalization procedure.

As miRNAs span a wide range of expression levels, median fold changes are a more accurate representation of miRNA expression values and are used throughout our miRNA analysis.

We used http://www.targetscan.org/ by Whitehead Institute for Biomedical Research (Cambridge, MA, USA) to check for conserved miRNA at the 3’UTR for genes affected. We also used the http://mirtarbase.mbc.nctu.edu.tw/index.html database to search miRNA target genes. This database collects data on miRNA-target interactions based on validated experiments.

### Statistical analyses

All statistical analyses were developed using R packages. MicroRNAs that showed signal in both tumor and normal tissue in at least 50% of cases were included in analyses presented here (Supplementary Table S1). Affymetrix gene expression array data obtained from different platforms were combined using the “matchprobes” package in R. For all Affymetrix array data (CEL files on all samples), after scan values were normalized using RMA as implemented in Bioconductor in R. For genes with more than one probe set, the mean gene expression was calculated. The GEO accession number of these array data is GSE44021 for mRNA at http://www.ncbi.nlm.nih.gov/geo/query/acc.cgi?acc=GSE44021 and GSE67268 for miRNA at http://www.ncbi.nlm.nih.gov/geo/query/acc.cgi?acc=GSE67268

Paired t-tests were used to identify differences in matched tumor/normal samples for mRNA expression. To find miRNAs with significant fold changes, we applied the Wilcoxon method to the fold change data in log10 scale with Bonferroni correction at 0.05, which resulted in a threshold *P*-value of 1.92E-04 (0.05/260 miRNAs tested). Spearman correlations were used to evaluate the association between expressions of miRNA and mRNA. Nearly six million (267 miRNAs × 22,277 mRNA probes = 5,947,959) Spearman correlations and their corresponding *P*-values were computed. To address the multiple testing problem here we used a Bonferroni corrected *P*-value cut off of 8.40E-09 (0.05/5,947,959 correlations tested) to select significant miRNA–target gene pairs. We also explored associations between miRNA and mRNA expression and clinical/pathological variables using Spearman analysis. For all evaluations presented here (including relating expression to survival), we used the miRNA signals (average delta Ct) or mRNA signals (average) for tumor:normal expressed as fold change ratios. For each miRNA or mRNA, we applied the Kaplan-Meier method to visualize differences and the Log-Rank test to statistically compare survival by expression levels divided as high versus low expression.

To further explore patterns of expression of miRNAs visually, we performed hierarchical clustering of data from miRNA expression by case. For this clustering, missing values were replaced by the median for each probe, and data were transformed to normalize their distribution. The R function ‘heatmap’ was used to generate the heatmap with the method set to ‘ward’ to calculate the distance used for the hierarchical clustering. We also evaluated the 11 demographic/clinicopathologic variables shown in Supplementary Table S2 in relation to different clusters of patients identified as shown in Supplementary Figure S1.

We used Cox proportional hazards regression models to evaluate survival as the hazard ratio (HR) for miRNA and gene expression fold change with adjustment of the four clinical variables age, gender, metastasis, and stage. We coded the fold change variables for miRNA and gene expression in two ways. First we assigned a single ordinal variable to represent each of the four quantile intervals (as 0, 1, 2, 3 to represent values in the ranges of 0 to 25%, 25 to 50%, 50 to 75%, and 75 to 100% of the distribution, respectively). Second, we created indicator variables for each of the four quartiles so that we could compare Q2, Q3, and Q4 separately to Q1 as the reference category.

## Results

### Patient information

Characteristics of the 113 total ESCC patients evaluated here are summarized (Supplementary Table S2) as follows: the median age for all patients was 57 years old with a range of 37 to 71 years; males predominated (62%); around half the patients reported tobacco use (52%) and alcohol use (50%); family history of UGI cancer was reported by nearly a third (30%) of cases; over three-quarter of tumors (80%) were grade 3, more than two-thirds (70%) were stage II, and metastatic disease was evident for nearly half the cases (46%).

### Identification of dysregulated miRNAs and mRNAs in ESCC

We performed both miRNA and mRNA arrays using tumor and matched normal tissues from 113 ESCC patients. 664 human miRNAs were investigated using the TaqMan® Low Density Array system on the expression values of each miRNA based on both tumor and normal tissues. 523 miRNAs showed signals in both tumor and normal in at least one case (due to tissue specificity, 114 miRNAs had no signal). In order to have sufficient numbers of cases with expression data for each miRNA, we required that at least half the patients express an miRNA in both tumor and normal tissue for it to be included in our analysis. This restriction reduced the number of miRNAs we analyzed here from 523 to 260.

Among the 260 miRNAs expressed in at least half the cases, 39 miRNAs showed dysregulation, defined here as a fold change of two or greater (ie, fold change < 0.50 for down-regulation or > 2.00 for up-regulation) and a *P*-value less than 0.05 after Bonferroni correction (in this case, 0.05/260 = *P* < 1.92E-04, including 28 miRNAs down-regulated and 11 up-regulated (Table 1). Table 1 also shows the frequency distribution of the 39 dysregulated miRNAs which indicates the dominant expression trend in cases. For example, expression of miR-375 was down-regulated in 82% of cases, while miR-196b was up-regulated in 84% of cases.

**Table 1:** Dysregulated miRNAs (FC ≤ 0.50 or FC ≥ 2.00, *P* < 1.92E-04; *N* = 39) in ESCC^a,b^

	**No.**	**miRNA**	**No.cases expressing miRNA**	**Median FC**	***P*-value**	**Frequency distribution of cases by FC category**		
						**FC ≤ 0.50**	**0.50 < FC < 2.00**	**FC ≥ 2.00**
**Down-regulated**	1	hsa-miR-375	90	0.02	9.38E-13	0.82	0.11	0.07
	2	hsa-miR-139-5p	112	0.14	7.30E-17	0.81	0.13	0.05
	3	hsa-miR-133a	113	0.19	1.18E-12	0.70	0.19	0.12
	4	hsa-miR-133b	111	0.20	1.52E-10	0.63	0.21	0.16
	5	hsa-miR-885-5p	86	0.27	2.26E-08	0.59	0.30	0.10
	6	hsa-miR-145	112	0.29	1.04E-10	0.63	0.24	0.12
	7	hsa-miR-486-5p	112	0.29	4.13E-10	0.70	0.16	0.14
	8	hsa-miR-204	99	0.30	7.02E-08	0.62	0.23	0.15
	9	hsa-miR-203	107	0.31	1.11E-04	0.58	0.23	0.19
	10	hsa-miR-30a*	113	0.31	6.96E-14	0.65	0.28	0.07
	11	hsa-miR-378*	104	0.34	1.05E-09	0.62	0.28	0.11
	12	hsa-let-7c	113	0.36	1.58E-09	0.63	0.24	0.13
	13	hsa-miR-23b	112	0.36	4.80E-11	0.59	0.32	0.09
	14	hsa-miR-125b	112	0.37	7.66E-09	0.54	0.32	0.13
	15	hsa-miR-422a	113	0.39	4.39E-10	0.58	0.31	0.12
	16	hsa-miR-149	113	0.40	2.64E-08	0.56	0.32	0.12
	17	hsa-miR-26b*	84	0.40	4.10E-07	0.60	0.27	0.13
	18	hsa-miR-30e*	113	0.40	1.88E-11	0.57	0.35	0.09
	19	hsa-miR-99a*	111	0.40	1.08E-07	0.59	0.24	0.16
	20	hsa-miR-328	108	0.41	1.07E-09	0.59	0.31	0.10
	21	hsa-miR-140-3p	113	0.44	2.89E-11	0.57	0.36	0.07
	22	hsa-miR-574–3p	112	0.45	9.99E-10	0.53	0.38	0.09
	23	hsa-miR-143	113	0.48	1.63E-07	0.51	0.35	0.13
	24	hsa-miR-378	113	0.48	1.05E-09	0.51	0.38	0.11
	25	hsa-miR-100	113	0.49	6.58E-07	0.50	0.39	0.11
	26	hsa-miR-150	113	0.49	1.64E-11	0.50	0.44	0.05
	27	hsa-miR-423-5p	103	0.49	1.87E-04	0.50	0.29	0.20
	28	hsa-miR-95	112	0.50	1.17E-06	0.50	0.38	0.12
**Up-regulated**	29	hsa-miR-183*	90	2.14	8.10E-07	0.12	0.33	0.54
	30	hsa-miR-106b	109	2.24	3.62E-08	0.10	0.39	0.51
	31	hsa-miR-708	110	2.29	3.46E-09	0.11	0.35	0.55
	32	hsa-miR-22	98	2.39	4.77E-06	0.18	0.26	0.56
	33	hsa-miR-639	83	2.44	1.30E-05	0.14	0.30	0.55
	34	hsa-miR-21*	110	2.69	3.64E-10	0.12	0.29	0.59
	35	hsa-miR-596	94	2.72	6.48E-06	0.17	0.27	0.56
	36	hsa-miR-130b	94	2.78	2.72E-08	0.11	0.31	0.59
	37	hsa-miR-124	100	2.98	7.20E-05	0.21	0.23	0.56
	38	hsa-miR-21	112	4.60	0.00E+ 00	0.02	0.21	0.78
	39	hsa-miR-196b	104	9.31	2.22E-16	0.06	0.11	0.84

^a^miRs sorted by ascending tumor/normal median fold change (FC)

^b^*P*-value threshold for multiple comparison adjustment is *P* < 1.92E-04 (0.05/260)

Hierarchical clustering was performed to characterize miRNA expression for all tumors and matched normal tissues. Heat maps showed similar patterns when using probe sets that had signals across all 113 samples in either 50% or 90% of the samples, so we report only results for probe sets with signals on at least half the samples. Here, we show that miRNAs (rows) cluster into two main groups with several sub-groups (Supplementary Figure S1). In the first main group (on the top), more than half of miRNAs show up-regulation (red), while the second main group (at the bottom) shows mainly down-regulation (green). The heat map also shows that patients (columns) can be divided into two main groups with either predominantly up- or down-regulated miRNAs. Heterogeneity in ESCC patients can be readily seen in the miRNA expression map. In addition, we evaluated several different clusters of patients identified in Supplementary Figure S1 in relation to the 11 demographic/clinicopathologic variables shown in Supplementary Table S2. Separately, we examined the 2 main clusters, the 3 main clusters, and the 4 main clusters, but none of these sets of clusters showed a relation to any of 11 demographic/clinicopathologic variables studied, including survival (all *P*-values > 0.10).

Gene expression (mRNA) was profiled on Affymetrix U133A chips and results analyzed with paired t tests. A total of 818 genes showed dysregulated gene expression between tumor and normal tissues, including 422 down-regulated and 396 up-regulated genes (a dysregulated gene was defined as one having a tumor:normal tissue expression fold change ratio of > 2.00 (or < 0.50) and a *P* < 2.24E-06, based on testing 22,277 probes (0.05/22,277 = 2.24E-06). The 10 most up-regulated genes were *MMP1*, *SPP1, COL11A1, COL1A1, POSTN, MMP12, MAGEA6, MAGEA3, COL1A2,* and *KRT17*; while the 10 most down-regulated genes were *CRISP3, CRNN, MAL, TGM3, CLCA4, SCEL, CRCT1, SLURP1, TMPRSS11E,* and *FLG.*

### Correlation between expression of miRNA and target genes in ESCC

Spearman analysis was applied for the correlation analysis between 267 microRNAs and all mRNAs expressed in both tumor and matched normal tissues (*n* = 22,277 mRNA probes, including all 818 dysregulated genes described above). Expression of 16 miRNAs showed correlation with expression of 195 genes at the *P* < 8.42E-09 level (Table 2 and Supplementary Table S3), including 153 positive correlations (rho range = 0.51 to 0.63) and 42 negative correlations (rho range = − 0.52 to − 0.56). For example, hsa-miR-320 is correlated with expression of two genes, and showed both positive (rho = 0.51 with *ACOX2* under expression) and negative (rho = − 0.54 with *EZH2* over expression) correlations. Taken together, these results indicate that one miRNA can target multiple genes and execute positive or negative effects on the expression of these genes.

**Table 2:** Correlated miRNA - gene expression pairs in ESCC^a^

**No.**	**miRNA**	**miRNA fold change**^**b**^	**No. correlated genes**	**Correlated gene**	**Gene fold change**^**c**^	**Rho**	***P*-value**
1	has-miR-155	1.73	3	*PSMB9*	2.50	0.57	<10E-12
				*BTN3A3 /// BTN3A2*	1.70	0.55	6.80E-10
				*UBE2L6*	1.90	0.54	2.20E-09
2	hsa-miR-133a	0.19	2	*SLC2A1*	2.40	−0.52	4.50E-09
				*SLC6A8*	1.90	−0.52	7.10E-09
3	hsa-miR-135a*	0.89	1	*BC37295_3*	0.83	0.53	2.80E-09
4	hsa-miR-145	0.29	1	*SMC4*	1.20	−0.54	1.50E-09
5	hsa-miR-149	0.4	6	*IFI30*	2.40	−0.52	4.80E-09
				*PSME2*	1.70	−0.52	6.30E-09
				*GZMA*	1.50	− 0.52	5.40E-09
				*PSMB8*	1.80	− 0.54	6.00E-10
				*TRA@ /// TRD@*	1.10	− 0.54	1.30E-09
				*PSMB9*	2.50	−0.56	3.70E-12
6	hsa-miR-150	0.49	5	*CCR7*	1.10	0.57	0
				*IGHM*	0.94	0.54	1.10E-09
				*SELL*	1.30	0.52	4.70E-09
				*CCL19*	0.81	0.52	6.70E-09
				*MS4A1*	1.10	0.52	5.80E-09
7	hsa-miR-200b	1.11	7	*GPR116*	1.10	−0.52	7.40E-09
				*DMD*	0.58	−0.53	4.90E-09
				*IL33*	0.61	−0.53	2.60E-09
				*RFTN1*	1.20	−0.53	4.30E-09
				*PPARGC1A*	0.71	−0.53	4.70E-09
				*GPM6A*	0.68	−0.54	1.50E-09
				*GEM*	0.80	−0.56	1.40E-10
8	hsa-miR-203	0.31	146	*DSG3*	0.45	0.63	<10E-12
				*CST6*	0.35	0.62	<10E-12
				*IL1RN*	0.16	0.62	<10E-12
				*MAP3K9*	0.50	0.62	<10E-12
				*GLTP*	0.28	0.62	<10E-12
				*AIM1L /// FLJ38020*	0.29	0.62	<10E-12
				*PPL*	0.17	0.61	<10E-12
				*SERPINB13*	0.19	0.61	<10E-12
				*FBXO34*	0.57	0.61	<10E-12
				*EVPL*	0.29	0.60	<10E-12
				*SPRR1B*	0.43	0.60	<10E-12
				*SERPINB13*	0.22	0.60	<10E-12
				*SPRR1A*	0.27	0.60	<10E-12
				*RAPGEFL1*	0.54	0.60	<10E-12
				*ZNF750*	0.29	0.60	<10E-12
				**(for full set of genes correlated with miR-203, see Supplementary Table****S3****)**			
9	hsa-miR-205	0.89	5	*F12*	1.10	0.57	<10E-12
				*STX6*	1.10	0.54	1.30E-09
				*HSPA4*	0.85	0.52	6.90E-09
				*MGC24039*	0.97	−0.52	5.60E-09
				*PFAAP5*	0.90	−0.53	3.50E-09
10	hsa-miR-214	1.17	3	*CHAF1A*	1.40	−0.52	4.70E-09
				*EZH2*	2.10	−0.54	1.60E-09
				*TMPO*	1.50	−0.54	8.50E-10
11	hsa-miR-224	1.56	1	*F12*	1.10	0.53	2.00E-09
12	hsa-miR-320	0.51	2	*ACOX2*	0.50	0.51	6.60E-09
				*EZH2*	2.10	−0.54	4.40E-10
13	hsa-miR-375	0.02	2	*IL1RN*	0.16	0.59	9.90E-10
				*PSG3*	0.62	0.58	1.40E-09
14	hsa-miR-574–3p	0.45	5	*ADD1*	0.72	0.53	2.30E-09
				*GAS7*	0.52	0.52	7.00E-09
				*EZH2*	2.10	−0.52	7.80E-09
				*TMPO*	1.50	−0.52	5.60E-09
				*C13orf34*	1.90	−0.52	4.80E-09
15	hsa-miR-650	0.98	15	*LOC91316*	1.50	0.64	<10E-12
				*POU2AF1*	1.70	0.60	<10E-12
				*CTA-246H3.1*	1.80	0.58	<10E-12
				*MGC29506*	1.30	0.58	<10E-12
				*CD79A*	1.00	0.55	2.50E-10
				*IGHA1 /// IGHG1 /// IGHG3 /// IGHM*	1.20	0.55	5.90E-10
				*IL8*	1.60	0.54	2.10E-09
				*IVD*	1.60	0.54	1.20E-09
				*CXCL13*	2.80	0.53	3.20E-09
				*IGL@*	2.30	0.53	3.70E-09
				*IGKC /// NTN2L /// GJB6*	2.40	0.53	2.60E-09
				*IGL@ /// IGLV4–3 /// IGLV3–25 /// IGLV2–14 /// IGLJ3*	2.30	0.53	3.10E-09
				*IGL@ /// IGLV4–3 /// IGLV3–25 /// IGLV2–14*	2.10	0.53	5.10E-09
				*HLA-C*	2.00	0.52	6.30E-09
				*IGL@ /// IGLV3–25 /// IGLV2–14 /// IGLJ3*	2.10	0.52	5.90E-09
16	hsa-miR-99b	0.79	1	*SMC4*	1.20	−0.52	4.40E-09

^a^*P*-value threshold for multiple comparison adjustment = *P* < 8.40E-09 (0.05/5,947,959)

^b^median miRNA fold change

^c^mean gene expression fold change

### Clinicopathological factors and miRNA expression in ESCC

Spearman analysis was also performed for associations between the various clinicopathological factors and 260 miRNAs, including metastasis (no vs yes), tumor grade (grade 1 and 2 vs grade 3 and 4), and tumor stage (stage I and II vs III and IV).

Twenty-six miRNA expressions were correlated with one of the three clinical phenotypes we evaluated at the level of nominal significance (*P* < 0.05; Supplementary Table S4), although none of the correlations was significant after adjustment for multiple comparisons (Bonferroni threshold *P* < 1.92E-04). Nine miRNAs correlated with the presence of metastasis (eg, miR-142-3p: FC 1.51, rho 0.28, *P* = 3.90E-03), seven with higher tumor grade (eg, miR-124a-3p: FC 0.76, rho − 028, *P* = 9.60E-03), and 10 with higher tumor stage (eg, miR-93*: FC 2.29, rho 0.26, *P* = 5.80E-03). These correlations were all moderate in magnitude, ranging from 0.19 to 0.28, and the fold changes observed were similarly modest, except for eight which exceeded twofold differences (six with FC < 0.50 and two with FC > 2.00). No overlapped miRNA was seen in the three categories. Taken together, we found no striking or clear-cut associations between miRNA expression and the clinicopathological features studied here.

### Cox model analysis of associations between 39 dysregulated miRNAs and survival in ESCC

We analyzed the expression of 39 dysregulated miRNAs with survival using Cox models with adjustment for age, gender, metastasis, and tumor stage (Table 3). Only two of these 39 miRNAs were associated with survival (nominal *P* < 0.05), including miR-30e* (HR = 0.76, 95% CI 0.61–0.95, *P* = 0.0179) and miR-124 (HR = 0.79, 95% CI 0.62–1.00), *P* = 0.0459).

**Table 3:** Survival by miRNA expression for 39 dysregulated miRNAs (from Table 1)^a,b,c,d^

**No.**	**miRNA**	**HR**	**95% CI**	***P*-value**
***1***	hsa-miR-30e*	***0.76***	***0.61–0.95***	***0.0179***
***2***	***hsa-miR-124***	***0.79***	***0.62–1.00***	***0.0459***
3	hsa-miR-22	1.22	0.95–1.56	0.1166
4	hsa-miR-140-3p	0.84	0.67–1.05	0.1303
5	hsa-miR-143	0.85	0.68–1.06	0.1487
6	hsa-miR-150	0.85	0.67–1.08	0.1924
7	hsa-miR-106b	1.16	0.92–1.45	0.2022
8	hsa-miR-203	1.15	0.92–1.44	0.2197
9	hsa-miR-596	0.87	0.69–1.09	0.2285
10	hsa-miR-30a*	0.88	0.71–1.09	0.2389
11	hsa-miR-23b	0.88	0.70–1.10	0.2638
12	hsa-miR-183*	1.15	0.89–1.48	0.2763
13	hsa-miR-204	0.88	0.69–1.12	0.2852
14	hsa-miR-486-5p	0.89	0.69–1.13	0.3295
15	hsa-miR-196b	0.89	0.71–1.13	0.3435
16	hsa-miR-145	0.90	0.73–1.12	0.3544
17	hsa-miR-133b	0.90	0.72–1.12	0.3565
18	hsa-miR-133a	0.90	0.72–1.13	0.3815
19	hsa-miR-26b*	0.89	0.68–1.18	0.4180
20	hsa-miR-21*	1.09	0.87–1.37	0.4533
21	hsa-miR-885-5p	1.08	0.85–1.38	0.5286
22	hsa-miR-130b	1.08	0.84–1.39	0.5384
23	hsa-miR-639	1.09	0.83–1.43	0.5404
24	hsa-miR-21*	1.07	0.86–1.34	0.5411
25	hsa-miR-423-5p	1.07	0.84–1.37	0.5707
26	hsa-miR-95	0.94	0.74–1.19	0.5844
27	hsa-miR-708	0.95	0.75–1.20	0.6622
28	hsa-miR-378*	1.05	0.84–1.32	0.6677
29	hsa-let-7c	0.95	0.76–1.20	0.6779
30	hsa-miR-328	0.95	0.74–1.21	0.6869
31	hsa-miR-574–3p	0.95	0.75–1.21	0.6977
32	hsa-miR-139-5p	0.96	0.77–1.20	0.7210
33	hsa-miR-375	1.04	0.81–1.33	0.7731
34	hsa-miR-149	0.97	0.76–1.22	0.7813
35	hsa-miR-125b	0.97	0.77–1.23	0.8095
36	hsa-miR-99a*	1.01	0.81–1.26	0.9020
37	hsa-miR-422a	0.99	0.79–1.23	0.9074
38	hsa-miR-100	1.01	0.80–1.26	0.9548
39	hsa-miR-378	1.00	0.80–1.26	0.9770

^a^miRNA expression modeled as tumor/normal fold change using ordinal variable (0,1,2,3)

^b^miRNAs shown in ascending order by *P*-value

^c^Cox proportional hazards models adjusted for age, gender, metastasis, stage

^d^Associations *P* < 0.05 are bolded and italicized

**Fig. 1:**
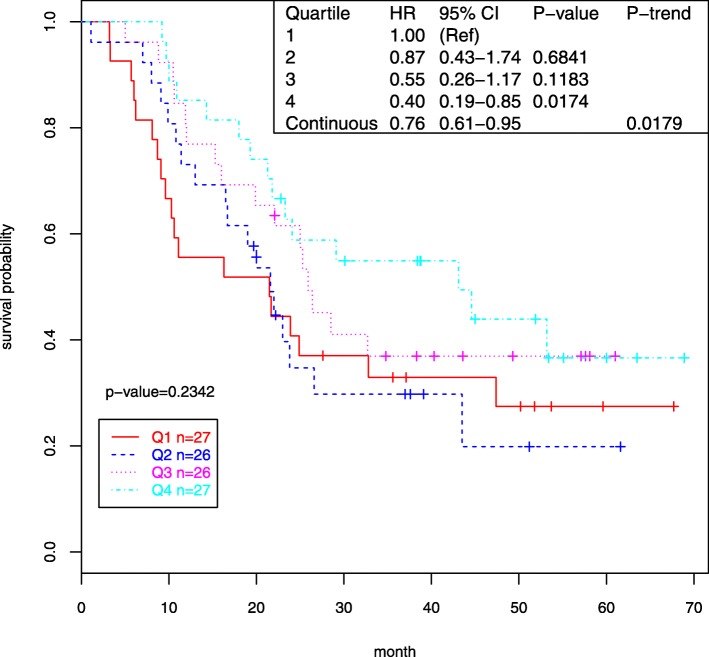
ESCC case survival by miR-30e* expression (Kaplan-Meier plot, Cox regression)

**Fig. 2:**
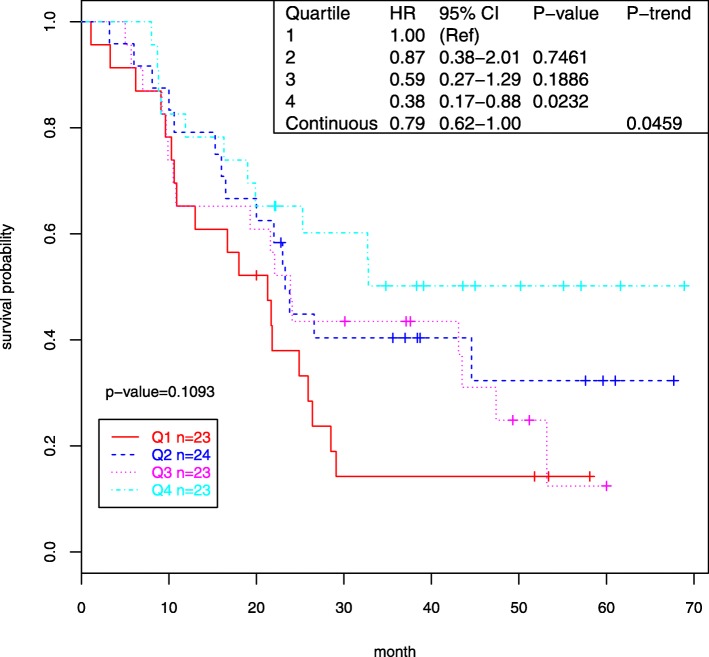
ESCC case survival by miR-124 expression (Kaplan-Meier plot, Cox regression)

The association between expression of these two miRNAs and survival was further analyzed by quartiles in Cox models. For both miRNAs, results showed that patients whose expression was in the highest quartile had substantially improved survival compared to patients in the lowest quartile (60% better for miR-30e* and 62% better for miR-124; Figs. 1 and 2, respectively). These differences represent improvements in median survival for patients in the highest quartile of miR-30E* over the lowest quartile of 10.4 months (21.4 months for quartile 1 vs 31.8 months for quartile 4) and of 9.4 months (24.6 months for quartile 1 vs 34.0 months for quartile 4) for miR − 124. Although neither of these survival associations withstood adjustment for multiple comparisons, the magnitude of the improvement in survival observed with increased expression of these miRNAs suggests that both miRNAs should be evaluated further in relation to prognosis.

### Cox model analysis of associations between 16 miRNAs correlated with gene expression and survival in ESCC

While the expressions of 16 miRNAs were identified as significantly correlated with expression of 195 genes, none of these miRNAs was significantly associated with survival after adjustment for age, gender, metastasis, and tumor stage using Cox models (all *P* > 0.05, Supplementary Table S5).

### Cox model analysis of associations between gene expression (mRNA) and survival in ESCC

We also investigated associations between the 195 genes (395 probes) that were significantly associated with miR expression (as shown in Table 2) and survival. Expressions of eight genes (nine probes) (*NF1, ASXL1, HSPA4, TGOLN2, BAIAP2, EZH2, CHAF1A, SUPT7L*) were associated with survival at the nominal significance level (*P* < 0.05) in Cox models adjusted for age, gender, metastasis, and stage (Supplementary Table S6). Further analyses of the nine probes (eight genes) with mRNA expression modeled as quartiles are shown in Table 4 and graphically as Kaplan-Meier plots in Supplementary Figure S2. The magnitude of the HR for persons in the highest quartile of expression was greatest for *NF1* (HR = 0.30); this translated into a median survival improvement of 11.1 months (for Q4 vs Q1). Median survival differences for persons in the highest vs lowest quartiles of gene expression were largest for *EZH2* and *CHAF1*A at − 18.3 and − 20.1 months, respectively.

**Table 4:** Survival by mRNA expression for 9 probes correlated with miRs in ESCC (from Supplementary Table S6)^a,b,c^

**No.**	**Gene/Probe**	**Quartile**	**HR**	**95% CI**	***P*-value**	**P-trend**	**Log-Rank *P*-value**
1	*NF1*/204323_x_at	1	1.00	(Ref)			
		2	0.93	0.45–1.93	0.8504		
		3	0.46	0.19–1.08	0.0748		
		***4***	***0.30***	***0.13–0.71***	***0.0062***		
						***0.0037***	0.0805
2	*ASXL1*/212237_at	1	1.00	(Ref)			
		2	0.90	0.44–1.83	0.7743		
		3	0.42	0.18–0.95	0.0371		
		4	0.46	0.20–1.06	0.0690		
						***0.0094***	0.1116
3	*HSPA4*/208814_at	1	1.00	(Ref)			
		***2***	***2.40***	***1.03–5.61***	***0.0433***		
		3	1.94	0.77–4.91	0.1610		
		**4**	***2.84***	***1.29–6.29***	***0.0099***		
						***0.0162***	***0.0139***
4	*TGOLN2*/212043_at	1	1.00	(Ref)			
		2	0.86	0.40–1.86	0.7014		
		3	0.57	0.26–1.26	0.1664		
		***4***	***0.35***	***0.14–0.86***	***0.0221***		
						***0.0223***	0.2394
5	*BAIAP2*/207832_at	1	1.00	(Ref)			
		2	0.69	0.36–1.34	0.2766		
		3	0.61	0.31–1.21	0.1588		
		4	0.51	0.22–1.15	0.1043		
						***0.0245***	0.0823
6	*EZH2*/203358_s_at	1	1.00	(Ref)			
		2	1.36	0.61–3.01	0.4540		
		***3***	***3.55***	***1.55–8.12***	***0.0027***		
		***4***	***2.27***	***1.01–5.11***	***0.0468***		
						***0.0247***	***0.0100***
7	*CHAF1A*/214426_x_at	1	1.00	(Ref)			
		***2***	***2.30***	***1.00–5.30***	***0.0499***		
		***3***	***2.65***	***1.18–5.92***	***0.0177***		
		***4***	***2.56***	***1.17–5.61***	***0.0190***		
						***0.0253***	0.0652
8	*TGOLN2*/212040_at	1	1.00	(Ref)			
		2	1.24	0.62–2.50	0.5435		
		3	0.55	0.25–1.25	0.1555		
		***4***	***0.35***	***0.13–0.95***	***0.0393***		
						***0.0258***	0.2537
9	*SUPT7L*/201836_s_at	1	1.00	(Ref)			
		2	1.09	0.47–2.52	0.8355		
		3	0.71	0.34–1.51	0.3746		
		4	0.42	0.17–1.01	0.0535		
						***0.0329***	0.4634

^a^mRNA expression modeled as tumor/normal mRNA expression fold change by quartile of expression

^b^Genes/probes shown in ascending order by *P*-trend value

^c^Models adjusted for age, gender, metastasis, stage

## Discussion

MicroRNAs (miRs) play an important central role in regulating the stability and expression of messenger RNA. To our knowledge, the present study is the largest to date to characterize genome-wide expressions of miRs and mRNAs in matched tumor/normal tissues from ESCC patients and relate those expressions to prognosis.

We identified 39 miRs that showed significant dysregulation in ESCC, including 11 up- and 28 down-regulated. Some of these miRNAs have been reported in cancers before, including ESCC (e.g., miR-143, miR-145, miR-196b, and miR-375). Among the dysregulated miRNAs identified, miR-196b showed the greatest up-regulation (FC 9.3) while miR-375 had the greatest down-regulation (FC 0.02). Over-expression of miR-196b has been previously described in ESCC, in pancreatic and gastric cancers, and in leukemia [11, 24–26]. This phenomenon, in which the same miRs are dysregulated in different cancers, suggests that these miRs are common regulators in human tumorigenesis. Interestingly, miR-375 was also dysregulated in esophageal adenocarcinoma (EAC), but there it was markedly up-regulated [27] as opposed to down-regulated as we observed here in ESCC and as been has reported by others in ESCC [12]. It is possible that the role of miR-375 in cancer has tissue and tumor specificity [28]. Overall, miR-375 appears to function as a tumor suppressor in ESCC but as an oncogene in EAC. Although miR-375 was not related to prognosis in ESCC patients in our study, lower expression of miR-375 was associated with poorer prognosis in several prior studies [13, 29]. Whether or not miR-375 is associated with survival, its extreme under-expression in ESCC suggests it merits further study as a potential early disease detection biomarker.

Many studies have identified numerous dysregulated miRs in various cancers. However, whether these dysregulated miRs influence gene targets in tumors is unclear. To better understand the associations between expression levels of miRs and gene targets, we performed genome-wide expression of miRs and mRNA using patient-matched tumor and normal tissues. We identified 16 miRs whose expressions correlated with gene expression (after Bonferroni correction), including six miRs whose tumor:normal expression FCs were < 0.50. For example, decreased expression of miR-133a (FC 0.19) correlated with up-regulation of *SLC2A1* (Solute Carrier Family 2 Member 1) (FC 2.40). This gene encodes a major glucose transporter in the mammalian blood-brain barrier. Lazar et al. reported increased expression of this gene in some malignant tumors and suggested a role for glucose-derivative tracers to detect in vivo thyroid cancer metastases by positron-emission tomography scanning [30]. On the other hand, decreased expression of miR-203 (FC 0.31) was associated with down-regulation of several genes, including *PPL***(**Periplakin**)** (FC 0.17) and *EVPL* (Envoplakin) (FC 0.29). The *EVPL* gene encodes a member of the plakin family of proteins that form components of desmosomes and the epidermal cornified envelope. This gene is located in the tylosis esophageal cancer locus on chromosome 17q25, and its deletion is associated with both familial and sporadic forms of ESCC [31]. *PPL* is an important paralog of the *EVPL* gene and both *EVPL* and *PPL* were down-regulated, indicating that miR-203 can regulate expression of more than one gene in ESCC. These results suggest that some miRs may act as tumor suppressors (eg, miR-133a) while others function as oncogenes (e.g., miR-203) in ESCC.

We identified three miRs (miR-214, FC 1.17; miR-320, FC 0.50; and miR-574–3p, FC 0.45; Supplementary Table S1) that correlated with up-regulation of *EZH2* (FC 2.10 for all three of these miRs, Table 2), a gene related to survival (Table 4 and Supplementary Figure S2). *EZH2* is an epigenetic regulator of the polycomb group proteins with important functions in embryonic stem cell regulation. Varambally et al. reported that *EZH2* was over-expressed in prostate cancer and associated with under-expression of miR-101 [32, 33]. In our study, expression of miR-101 (median FC 1.2, range 0.005 to 79.7) was not correlated with expression of *EZH2*, but ESCC patients who over-expressed this gene had shorter survival (HR = 1.30, 95% CI 1.03–1.62, nominal *P* = 0.0247).

Although we found 16 miRs whose expression correlated with gene expression, the magnitude of the tumor:normal expression level ratios in 10 of these miRs was in the normal range (i.e., 0.50 < FC < 2.00). For example, miR-155 (FC 1.73) correlated with over-expression of *PSMB9* (FC 2.50), and miR-650 (FC 0.98) correlated with over-expression of *CXCL13* (FC 2.80). It seems clear that there are many factors that can influence gene expression beyond just the effect of miRs (e.g., DNA mutations, splice changes), and that widespread dysregulation of cancer-associated miRs is a complex phenomenon. Many of these miRs have been located downstream of major oncogenes and tumor suppressors that act as transcription factors [28]. Thus, paired studies on expression of both miRs and gene targets as well as other studies (e.g., somatic mutation, methylation) using the same samples may help us better understand the role these cancer-associated miRs play in human cancers.

miR expression has been associated with diagnosis, prognosis, and response to treatment in various cancers. The present study suggests a number of potential miRs that might serve as early detection or diagnostic markers. Essentially all 39 dysregulated miRs shown in Table 1 can be considered candidate early detection/diagnosis markers for ESCC, although the most attractive candidates among these are the miRs with the most extreme tumor/normal fold changes, particularly miRs with elevated FCs, since laboratory tests that measure increased levels are typically easier to develop and interpret than tests that measure decreased levels. The miRs identified with the most extreme FCs include miR-375 (FC 0.02), miR139-5p (FC 0.14), and miR-133a (FC 0.19) with the lowest FCs, and miR-196b (FC 9.31), miR-21 (FC 4.60), and miR-124 (FC 2.98) with the highest FCs. More study is required, however, to establish the true clinical usefulness of these miRs as early detection/diagnosis markers in ESCC, including evaluation in more cases as well as controls to affirm findings from the current research and address traditional screening test parameters (e.g, sensitivity, specificity, etc). At some point it will be necessary to evaluate these miRs in esophageal squamous epithelium in patients representing a spectrum of disease that includes normal, premalignancy, and early invasive disease to determine when miR expression changes occur in the ESCC carcinogenesis process. If blood levels of these miRs can be shown to reproducibly reflect levels in esophageal tissue, early detection screening and/or diagnosis might be reducible to a simple blood test.

This study also found evidence that expression of selected individual miRs might serve as prognosis markers in ESCC cases. Expressions of two miRs – miR-124 and miR-30e* – were both associated with survival in ESCC patients. miR-124 was up-regulated in ESCC patients (FC 2.98) and patients with higher expression levels lived longer, while miR-30e* was down-regulated (FC 0.40) but patients with lower expression levels also survived longer.

Based on findings from the current study, the most attractive individual miR for future development as a potential biomarker in ESCC appears to be miR-124. This is because miR-124 was markedly elevated in tumor compared to normal tissue (FC 2.98), making it attractive as a potential early detection/diagnosis marker. At the same time, expression of miR-124 was also associated with survival, suggesting that it may serve as a prognosis marker as well.

Hierarchical clustering identified collections of ESCC cases whose miR expression profiles grouped them together. Unfortunately, survival experience for patients in these different groupings did not differ.

In addition to the miR-30* and miR-124 associations with survival noted above, expression of eight genes (9 different mRNA probes) were also nominally associated with survival. Additional work is needed to establish if the observed associations with survival in ESCC for these mRNAs are real or just false positives. Further, inherent instability in mRNAs makes their practical use as biomarkers very difficult, so, in addition to further validating the use of these mRNAs as prognosis markers, efforts to translate assessment of mRNA expression into gene-specific protein expression assays that can be readily applied in clinical labs (e.g., through immunohistochemistry tests) are also needed.

Our identification of both miRs and genes whose expressions separately appear to relate to survival in ESCC patients suggests that further exploration of models employing miR and gene markers together might lead to markers or signatures with improved predictive performance. Thus, integrating the data from different sources initially generated to inform on the biologic role of these small molecules might also find clinical relevance as markers for early detection, diagnosis, or prognosis.

## Conclusions

Using genome-wide platforms in tumor and normal tissues we identified 39 miRs with dysregulated expression in ESCC patients. Combining these miR data with genome-wide RNA expression data on the same patients, we further determined that expression of 16 miRs strongly correlated with RNA expression in 195 genes. We found both miRs and RNAs whose expressions showed suggestive associations with clinical characteristics and survival. Taken together, our findings provide insights into the expression of miRs and their relation to regulation of RNA targets in ESCC tumorigenesis, and suggest opportunities for the future development miRs and mRNAs as biomarkers for early detection, diagnosis, and prognosis in ESCC.

## Supplementary information

**Additional file 1: Table S1.** 260 miRNAs with tumor & normal tissue expression in ≥50% of cases.

**Additional file 2: Table S2.** Demographic and clinical characteristics of ESCC cases (*n* = 113).

**Additional file 3: Figure S1.** Heatmap of miRNAs dysregulated in at least half of ESCC cases.

**Additional file 4: Table S3.** Gene/probe expressions correlated with miR-203

**Additional file 5: Table S4.** Correlation of miRNA expression with clinical characteristics.

**Additional file 6: Table S5.** Survival by miR expression for 16 miRs correlated with gene expression (from Table 2).

**Additional file 7: Table S6.** Survival by mRNA expression for 395 probes (in 195 genes) correlated with miRs.

**Additional file 8: Figure S2.** Kaplan-Meier survival plots by quartile of RNA expression for 9 probes correlated with miRNAs (from Supplementary Table S6).

## Data Availability

The GEO accession numbers of the array data analyzed for this manuscript are GSE44021 for the mRNA data (available at http://www.ncbi.nlm.nih.gov/geo/query/acc.cgi?acc=GSE44021) and GSE67268 for the miRNA data (available at http://www.ncbi.nlm.nih.gov/geo/query/acc.cgi?acc=GSE67268). Clinical data analyzed in the current study are not publically available due to presence of identifiable patient information but are available from the corresponding author upon reasonable request.

## Abbreviations

CI: confidence interval
ESCC: esophageal squamous cell carcinoma
EAC: esophageal adenocarcinoma
FC: fold change
HR: hazard ratio
miR: miRNA = micro RNA
mRNA: messenger RNA
Q1: quartile 1
