# Supplementary figures and images for: Integrated analysis of genome-wide miRNAs and targeted gene expression in esophageal squamous cell carcinoma (ESCC) and relation to prognosis
Source: BMC Cancer. 2020 May 6;20:388. doi: 10.1186/s12885-020-06901-6 (PMC7201714; doi:10.1186/s12885-020-06901-6)

Suppl Fig S1

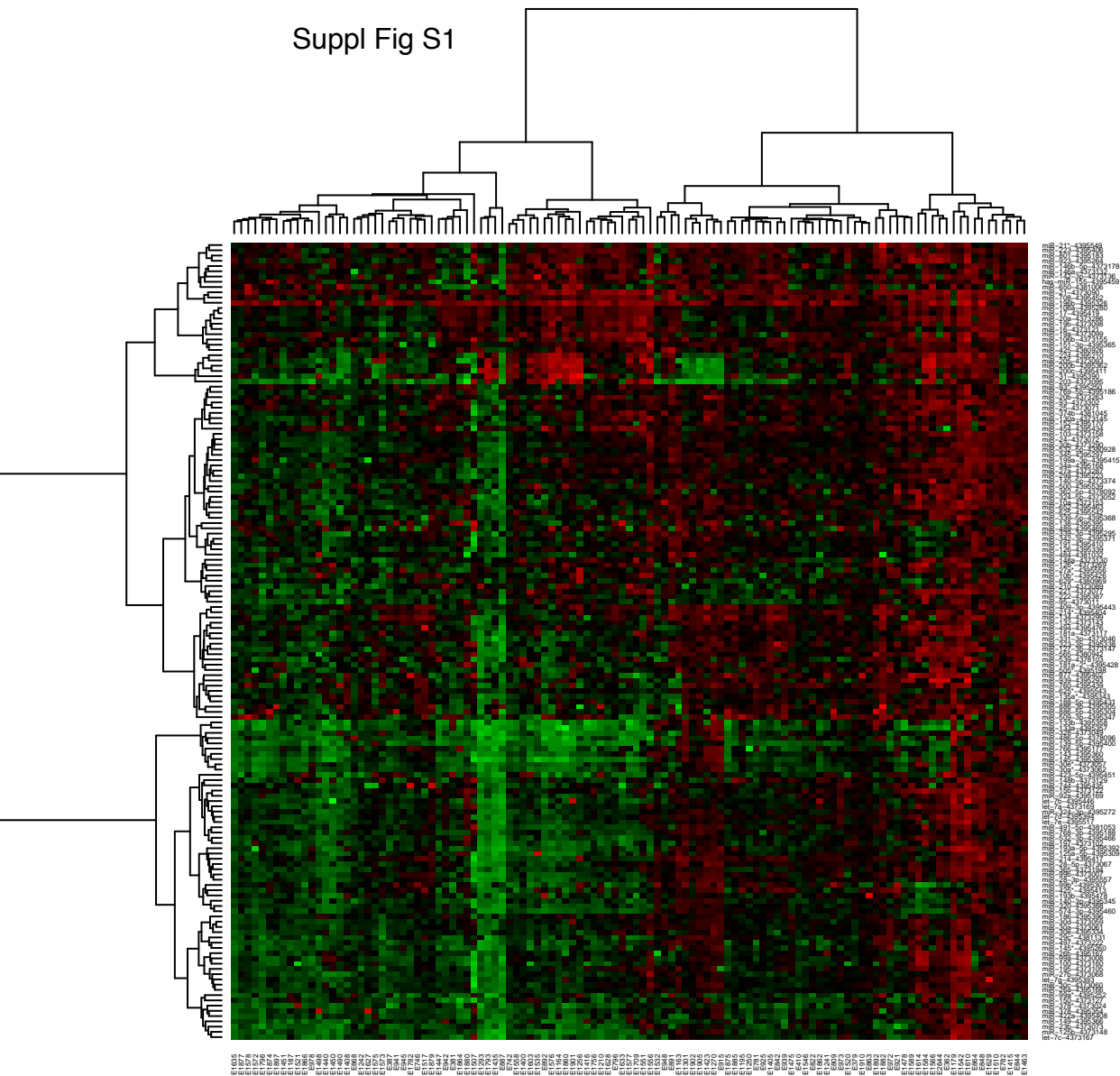

Supplement: Supplementary file 3 — Additional file 3: Figure S1. Heatmap of miRNAs dysregulated in at least half of ESCC cases. [file 12885_2020_6901_MOESM3_ESM.pdf]

Suppl Fig S 2

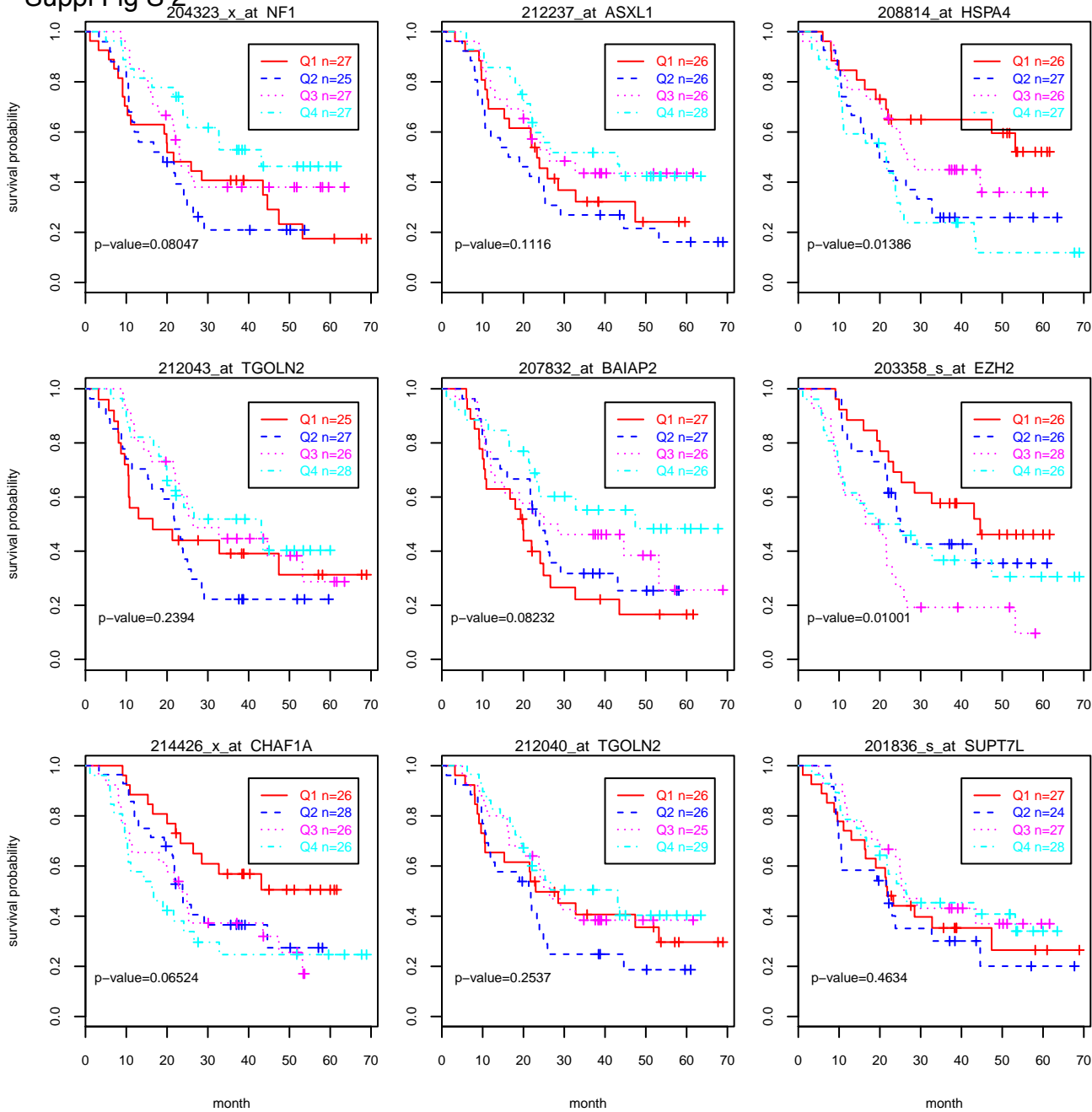

Supplement: Supplementary file 8 — Additional file 8: Figure S2. Kaplan-Meier survival plots by quartile of RNA expression for 9 probes correlated with miRNAs (from Supplementary Table S6). [file 12885_2020_6901_MOESM8_ESM.pdf]
